# Tislelizumab-induced type 1 diabetic ketoacidosis in a patient with small cell lung cancer: a case report

**DOI:** 10.3389/fonc.2025.1498701

**Published:** 2025-03-06

**Authors:** Jie Zhu, Wen-jie Wang

**Affiliations:** ^1^ Department of Oncology, Changzhou Traditional Chinese Medicine Hospital, Changzhou, China; ^2^ Department of Radio-Oncology, Suzhou Municipal Hospital, The Affiliated Suzhou Hospital of Nanjing Medical University, Suzhou, China

**Keywords:** tislelizumab, immune-related adverse event, type 1 diabetes mellitus, diabetic ketoacidosis, small cell lung cancer

## Abstract

This report presented a case of 71-year-old man diagnosed with extensive-stage small cell lung cancer (ES-SCLC) who developed type 1 diabetic ketoacidosis (DKA) after 3 cycles of tislelizumab plus chemotherapy for the first time. The patient had no history of diabetes mellitus (DM). According to medical history and laboratory examination, the case was definitely diagnosed new-onset type 1 diabetic ketoacidosis induced by tislelizumab, a kind of immune checkpoint inhibitor. Despite the incidence of immune checkpoint inhibitor-induced type 1 diabetes mellitus (ICI-T1DM) is rare, the development of ICI-T1DM, especially type 1 diabetic ketoacidosis is life-threating without blood glucose monitoring and insulin therapy. Early identification of hyperglycemia and C-peptide depletion, as well as routine blood glucose monitoring during ICI treatment is essential to avoid lethal endocrine immune-related adverse event (irAE).

## 1 Introduction

Small cell lung cancer (SCLC), an aggressive type of lung cancer with high mortality and poor outcome, accounts for 10-15% of all lung cancer cases (1). Approximately 80-85% of the patients are extensive-stage at diagnosis (2). Previously, platinum-based chemotherapy was traditional treatment for extensive-stage small cell lung cancer (ES-SCLC). However, despite high sensitivity to chemotherapy, the overall survival (OS) of ES-SCLC is 10 months and only approximately 7% cases survive beyond 5 years (3). Currently, with the emergence of immune checkpoint inhibitors (ICIs) in anti-cancer therapy, ICIs plus platinum-based chemotherapy has gradually taken over the first-line treatment in ES-SCLC. Tislelizumab is a kind of anti-programmed cell death-1 (anti-PD-1) antibody independently developed by China. Recently, RATIONALE-312 has claimed the efficacy and safety of tislelizumab plus platinum-etoposide chemotherapy as first-line treatment for ES-SCLC compared to chemotherapy (4). Moreover, BGB-A317-312, a multicenter randomized phase 3 clinical trial for tislelizumab plus platinum-based chemotherapy versus platinum-based chemotherapy, has been in progress.

Immune checkpoint inhibitor-induced type 1 diabetes mellitus (ICI-T1DM) is attributed to autoimmune destruction of pancreatic islet cells and characterized with absolute insulin deficiency. The occurrence of ICI-T1DM is approximately 1-2% among ICIs treatment (5). The median onset of ICI-T1DM is after 4.5 cycles of ICIs treatment (6). In a case report of Greene et al., a case of metastatic melanoma developed ICI-T1DM after 7 months of pembrolizumab treatment and lifelong insulin treatment was required (7). Daetwyler et al. have reported a case of metastatic renal cell carcinoma who developed ICI-induced diabetic ketoacidosis after accepting a combined of nivolumab and ipilimumab treatment for 4 months (8). Moreover, a case of advanced non-small cell lung cancer developed ICI-T1DM ketoacidosis after 4 weeks durvalumab treatment (9). ICI-T1DM in SCLC patients has been reported rarely. For instance, Huang et al. has reported a case of sintilimab-induced DKA (10) and Wen et al. has reported a case of durvalumab-induced T1DM (11) in SCLC. Moreover, in the latest RATIONALE-312 trial, 4 cases (2%) of T1DM was observed, and 3 cases (1%) were severe (grade ≥3) (4).

To the best of our knowledge, the present case was the first record of tislelizumab-induced T1DM in ES-SCLC. We hoped to draw a lesson from the present case and attach importance to blood glucose monitoring and health education to avoid lethal endocrine immune-related adverse event (irAE) in future clinical treatment of tislelizumab.

## 2 Case description

**Figure 1:**
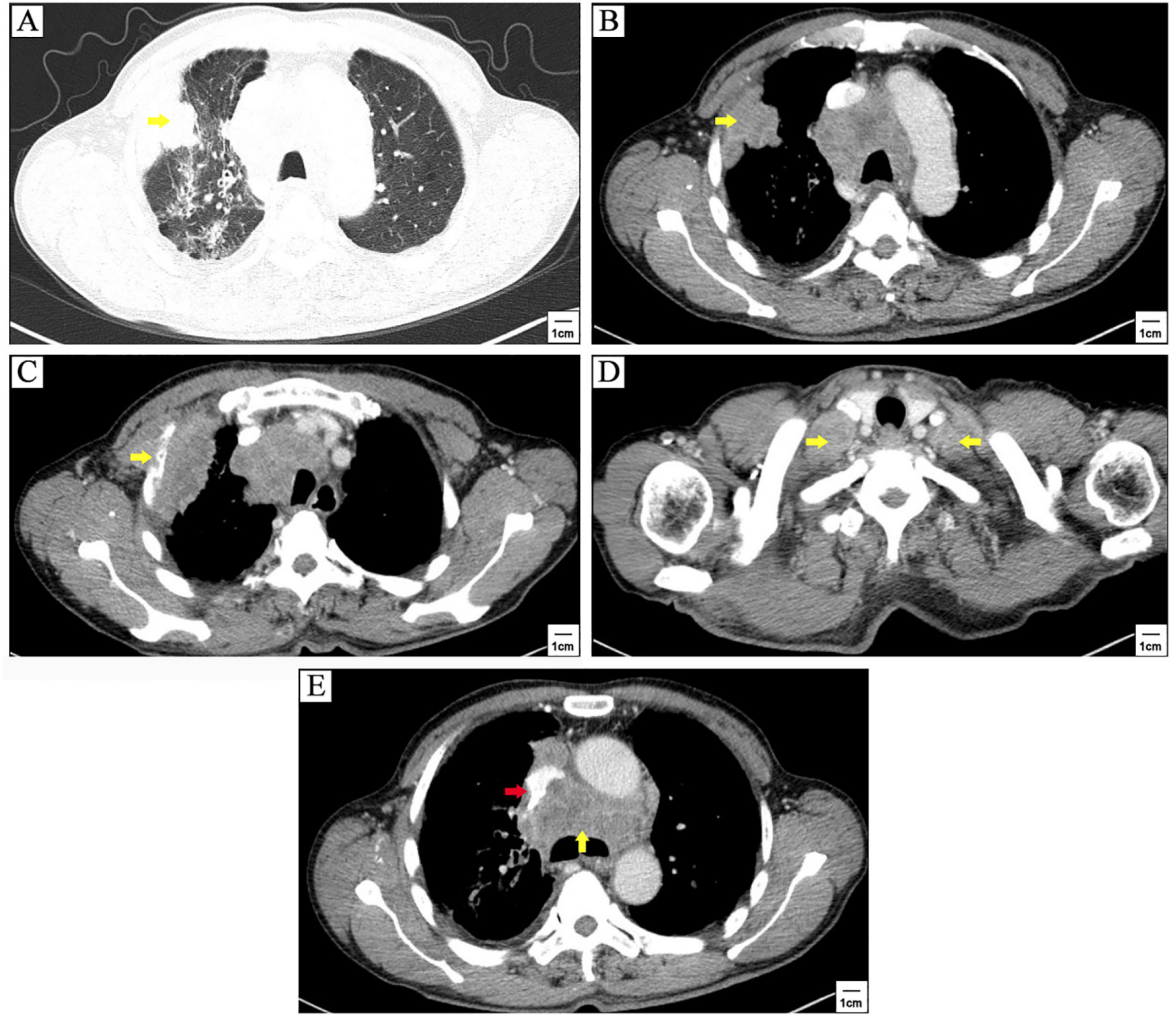
Enhanced computerized tomographic (CT) scanning at first referral. A subpleural tumor (79mm×37mm) in the upper lobe of the right lung (yellow arrowhead) **(A, B)**, with metastasis to rib (yellow arrowhead) **(C)**, lavicular region lymph nodes (yellow arrowheads) **(D)**, and mediastinal lymph nodes (yellow arrowhead) **(E)**, as well as superior vena cava obstruction (red arrowhead) **(E)**.

**Figure 2:**
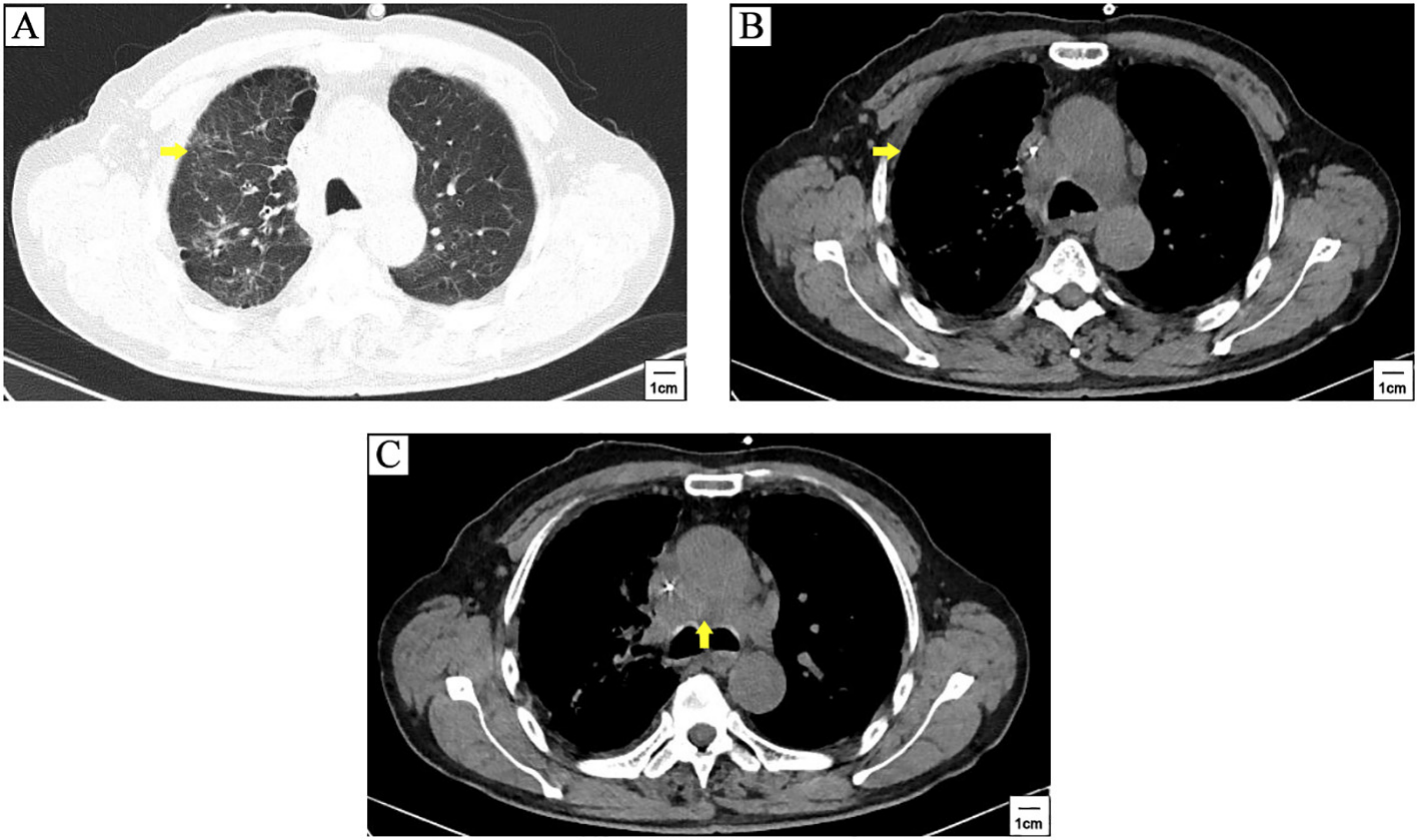
CT scanning after 3 cycles of tislelizumab plus cisplatin-etoposide treatment. A distinct regression of subpleural tumor (yellow arrowhead) **(A, B)** and mediastinal lymph nodes metastasis (yellow arrowhead) **(C)** was observed compared to pre-treatment CT scanning in **Figure 1**.

The patient was a 71-years-old man who visited local hospital for consistent cough and chest pain 5 months before the present admission. Enhanced computerized tomographic (CT) scanning showed a subpleural tumor (79mm×37mm) in the upper lobe of the right lung, with superior vena cava obstruction, as well as metastasis to rib, mediastinal lymph nodes, bilateral supraclavicular lymph nodes and bilateral subclavian lymph nodes (**Figure 1**). Cerebral magnetic resonance imaging (MRI) showed no brain metastasis. Further supraclavicular lymph node biopsy has confirmed metastatic small cell lung cancer (SCLC). The patient received 3 cycles of tislelizumab plus cisplatin-etoposide treatment. CT scanning observed a distinct regression of subpleural tumor and mediastinal lymph nodes (**Figure 2**). No brain metastasis was detected by CT scanning.

The patient was 170 cm tall and weighed 55 kg, with a BMI of 19.0. The patient had a long history of smoking and drinking. However, he had no past medical or family history of DM. His fasting plasma glucose (FPG) before tislelizumab treatment was 5.4mmol/L (normal range: 3.9-6.1mmol/L). The patient was less-educated and made a living from farming. Hence, he barely took biochemical test after a course finished and no after discharge blood tests were provided, so the only approach to obtain his glucose data was routine blood tests before the next course started and re-examination during hospitalization. After the first course of tislelizumab injection, the FPG was 5.2mmol/L. Before the second course, the FBG was 5.36mmol/L. After the second course, the FPG was 4.76mmol/L. Before the third course, the FBG was 4.75mmol/L. After the third course, the FPG was 6.28mmol/L, suggesting impaired fasting glucose (IFG). Then, the patient failed to finish the treatment due to financial reasons. And then, no routine blood tests were taken and no glucose monitoring was proceeded. 107 days after the start of tislelizumab therapy, the patient was admitted to emergency room with fatigue, drowsiness, nausea and vomiting which lasting for 3 days, and diagnosed as DKA. Laboratory examinations at emergency room were as follow: His capillary blood glucose (CBG) was undetectable high. Blood tests showed venous blood glucose of 91.26mmol/L, HbA1c of 10.8% (normal range: 4-6%), fasting C-peptide of 0.19ng/mL (normal range: 1.1-4.4ng/mL). Insulin autoantibody (IAA) showed negative. Urinalysis showed glucose 4+ and ketone body 2+. Arterial blood gas showed pH of 7.22, sodium of 126mmol/L, chlorion of 82mmol/L, potassium of 6.4mmol/L, HCO3^–^ of 15.80mmol/L, lactic acid of 2.7mmol/L, anion gap of 27.50mmol/L, and base excess of -11.7mmol/L. Blood biochemistry showed estimated glomerular filtration rate (eGFR) of 35.53mL/min (calculated by MDRD formula), urine nitrogen of 20.84mmol/L, and uric acid of 820µmol/L.

An insulin continuous infusion and infusion of saline solution was immediately started at the diagnosis of DKA. The level of CBG was tested per hour. Saline solution was changed to infusion of 5% glucose solution when CBG was reduced to 13.90mmol/L. The total fluid intake was controlled up to 4000-6000mL and urine output was monitored to prevent acute heart failure. The levels of electrolytes were monitored by arterial blood gas. Immediate CBG level after hospitalization was still undetectably high at 11:30am. CBG level was decreased to 30.4mmol/L at 16:30pm. After 10 hours, CBG of the patient has reduced to 10.9mmol/L at 21:30pm, and stabilized at 10-15mmol/L on the second day of hospitalization. No cerebral edema syndromes such as headache, dizziness, nausea, blurry vision and deteriorating conscious were observed in the whole treatment. The patient became clear in consciousness and started to eat on the third day of hospitalization. Arterial blood gas showed pH of 7.35, sodium of 161mmol/L, chlorion of 121mmol/L, potassium of 3.4mmol/L, HCO3^–^ of 25.80mmol/L, lactic acid of 2.5mmol/L, anion gap of 12.30mmol/L, and base excess of 2.2mmol/L. Blood biochemistry showed eGFR of 90.46mL/min, urine nitrogen of 6.1mmol/L, and uric acid of 438µmol/L. Rehydration of 5% glucose solution and intravenous diuretic were administered to prevent hypernatremia and hyperchloremia. Urinalysis showed that urinary glucose was 1+ and ketone body turned negative, whereupon continuous infusion of insulin was converted to subcutaneous injection of insulin aspart before each meal and insulin glargine before sleep. On the fifth day of hospitalization, when his CBG was stabilized by subcutaneous insulin treatment, 8.00 AM cortisol, thyroid function, fasting C-peptide, and 2-hour post-meal C-peptide were re-examined. His fasting C-peptide was 0.4ng/mL while 2-hour post-meal C-peptide was 0.41ng/mL. Test for IAA was negative. Thyroid function tests (TFTs) showed free triiodothyronine (FT3) of 3.08pmol/L (normal range: 3.1-6.8pmol/L), free thyroxine (FT4) of 18pmol/L (normal range: 12-22pmol/L), thyrotropin (TSH) of 0.031µmol/L (normal range: 0.27-4.2µmol/L).

**Figure 3:**
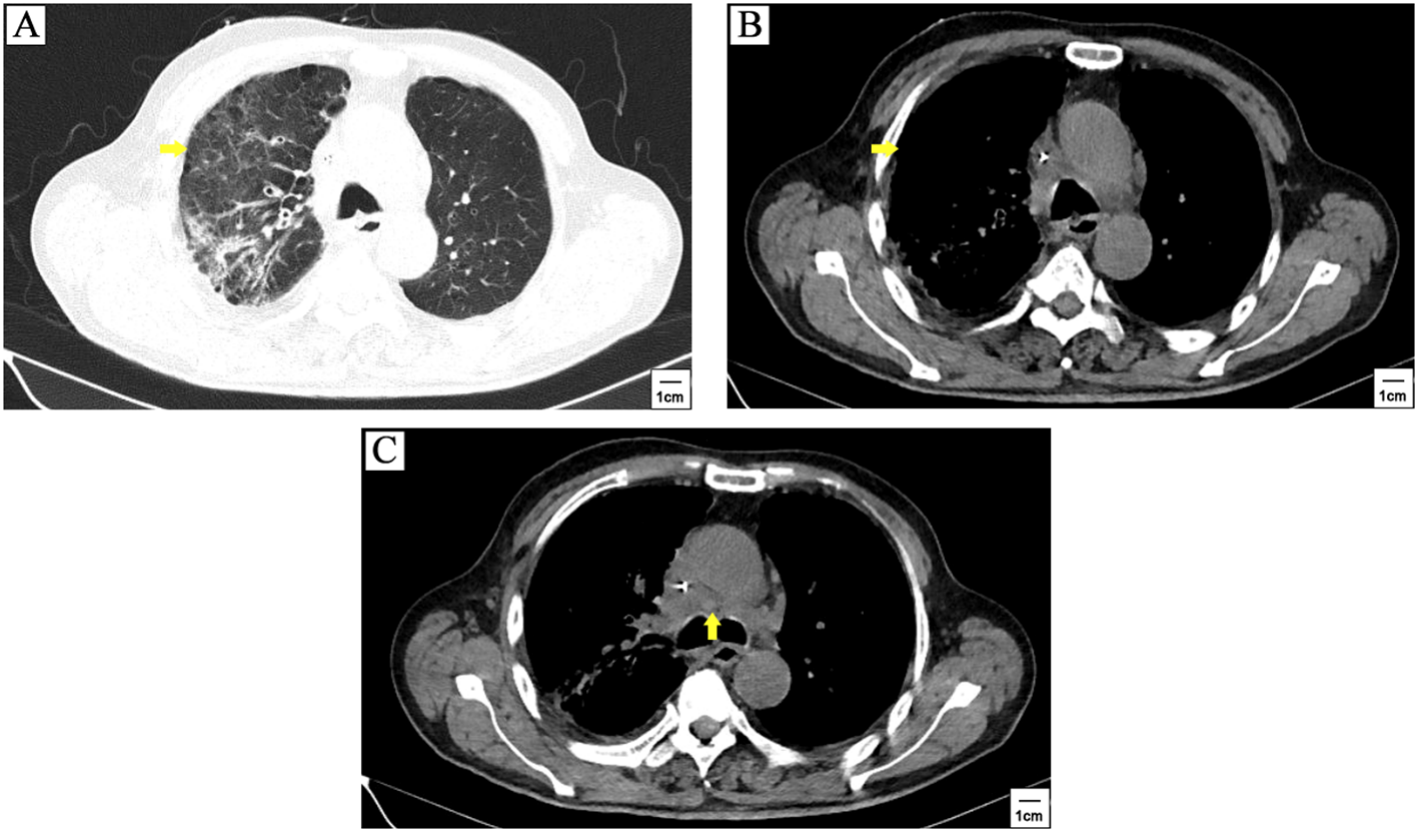
The patient quit anti-cancer treatment after 3 cycles of tislelizumab plus cisplatin-etoposide treatment. CT scanning showed stable disease when patient withdrew anti-cancer therapy for 2 months. The primary lesion (yellow arrowhead) **(A, B)** and mediastinal lymph nodes metastasis (yellow arrowhead) **(C)** was similar to the first follow-up.

**Figure 4:**
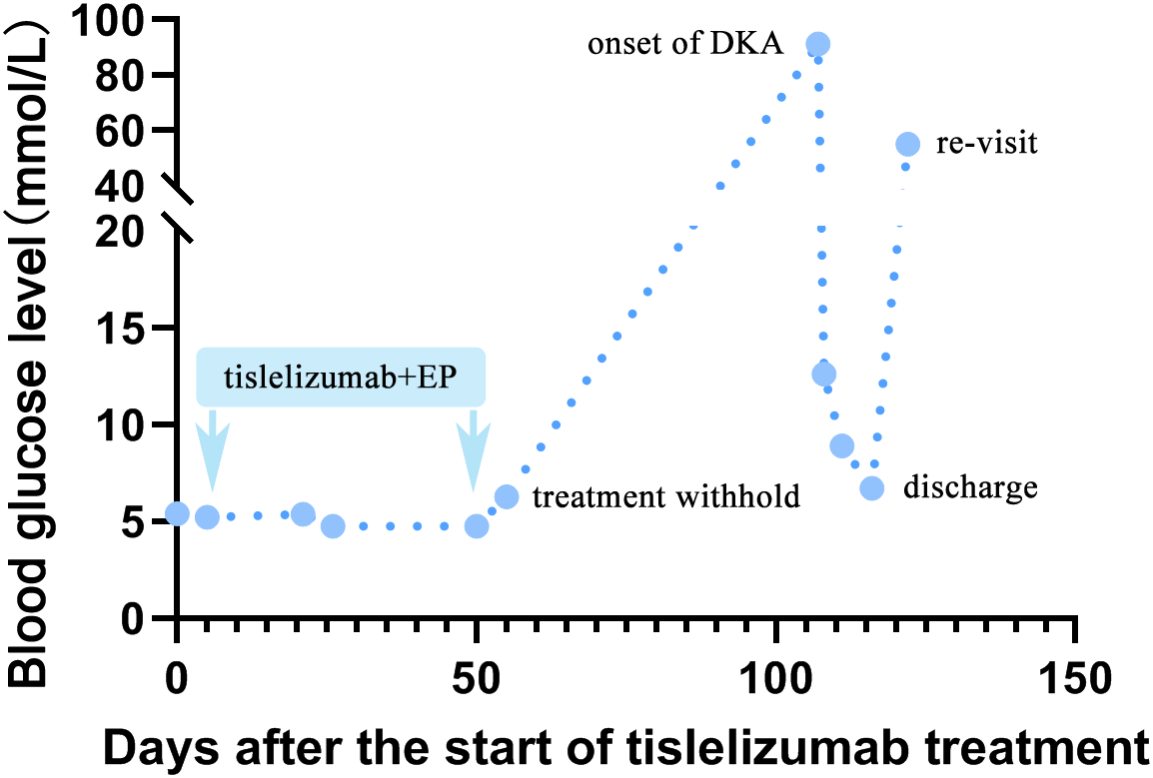
A line chart of blood glucose fluctuation. The fasting plasma glucose before tislelizumab treatment was among normal range. After administration of the third course of tislelizumab plus platinum-etoposide chemotherapy, an increase in FBG (6.28mmol/L) was observed on 56 days after the start of tislelizumab therapy, suggesting impaired fasting glucose (IFG). Then, the patient quit anti-cancer therapy. New onset of diabetic ketoacidosis (DKA) attacked the patient on 107 days after the start of tislelizumab therapy, with an undetectably high by peripheral blood and venous blood test showed glucose of 91.26mmol/L at emergency room. The patient got a stable glycemic control by subcutaneous insulin treatment at 10-15mmol/L during 10 days hospitalization. However, due to neglect of glucose monitoring and irregular insulin treatment, he re-visited our clinic for hyperglycemia (random venous glucose: 55.08mmol/L) just 6 days after discharge.

The patient got a stable glycemic control by subcutaneous insulin treatment and discharged on the tenth day of hospitalization. However, due to the neglect of glucose management, he re-visited our clinic for just 6 days after discharge. Venous blood tests showed a random glucose level of 55.08mmol/L. Chest CT scanning showed stable disease (SD) (**Figure 3**). The patient refused to take cerebral CT scanning as well as anti-cancer treatment once again. Considering the patient had poor compliance to insulin treatment, he was referred to endocrine department for better glycemic control. **Figure 4** illustrated the glucose fluctuation and treatment.

## 3 Discussion

With an incidence of 4-30%, IR-endocrinopathies are relatively frequent irAEs (12, 13). Primary hypothyroidism (6-9%) is the most common irAE, while ICI-T1DM (1-2%) is a rare but potential lethal irAE (5). In a meta-analysis involving 90 ICI-T1DM cases, the median onset time of ICI-T1DM was after 4.5 cycles of ICIs treatment, and the earliest case was 2.7 cycle. 70% of the cases were diagnosed as DKA. Autoantibody to islet cells was positive in 53% of the cases. Moreover, 24% of the cases were accompanied with thyroid disorders (6). Previous researches have revealed multiple molecular mechanisms of ICI-T1DM. For example, when ICIs enhances the anti-tumor immunity via inhibiting PD-1/PD-ligand 1 (L1) pathway, activated autoreactive T-cells contributed to the death of pancreatic β-cells by nitric oxide at the same time (14, 15). Moreover, animal experiment demonstrated that during anti-PD-L1 therapy, activated cytolytic interferon (IFN)-γ^+^CD8^+^ T-cells infiltrated into pancreas islets and induced the dedifferentiation of pancreatic β-cells, which leaded to rapid development of diabetes (16). Interestingly, a recent study reported an atezolizumab induced T1DM ketoacidosis in a SCLC patient with pre-existing type 2 diabetes mellitus (T2DM), suggesting that more attention should be paid in patients with pre-existing glucose intolerance (17).

The diagnosis of T1DM is hyperglycemia with persistent low C-peptide level or positive autoantibodies. The pathogenesis of T1DM can be divided into three stages that relate to the detection of autoantibodies and progress to β­-cells destruction, dysglycemia, and symptoms associated with hyperglycemia. Stage 1 is characterized by the presence of autoantibodies and no symptoms of hyperglycemia were observed. At stage 2, both autoantibodies and dysglycemia were observed, for example, IFG and impaired glucose tolerance (IGT). Symptomatic T1DM appears at stage 3 (18). DKA, a life-threatening acute complication of T1DM, is characterized with hyperglycemia, acidosis and ketosis. The clinical symptoms of DKA include fatigue, polydipsia, fatigue and vomiting (6). In the research of Baden et al., fulminant type 1 diabetes (FT1D) accounted for 50% of all ICI-T1DM cases (19). Patients with FT1D usually develop DKA within 1 week after the onset of hyperglycemia, with a drastic decline in plasma C-peptide level at the diagnosis of DKA (20). FT1D is the most common manifestion of ICI-T1DM in western counties (21, 22). In the present case, after 3 cycles of tislelizumab plus chemotherapy, the patient showed IFG, indicating a stage 2 T1DM process. However, the follow-up treatment and routine blood tests including glucose, C-peptide and HbA1c were suspended for nearly 2 months, just then, DKA suddenly attacked the patient. Thus, we failed to detect the early evidence of hyperglycemia and new onset of ICI-T1DM. In retrospect of the diagnosis and treatment of the present case, despite the negative result of IAA, hyperglycemia with persistence low level of fasting and C-peptide definitely supported the diagnosis of ICI-T1DM. Throughout the whole treatment, what is more worthy of reflection is insufficient consideration to elevated fasting blood glucose and lack of multipoint monitoring of blood glucose such as pre- and postprandial glucose. As the gold standard for evaluation of diabetes control, HbA1c gives an estimate of the blood sugar levels of an individual over the last three months. Duration of abnormal glucose revealed by HbA1c was consisted with the ICI treatment span. Thus, increased HbA1c in this case strongly suggested glycometabolic dysfunction.

Additionally, his TFTs before tislelizumab therapy was normal, while a distinct decrease of TSH and FT3 was observed, suggesting accompanied central hypothyroidism in the present case. Nevertheless, low T3, FT3 and TSH concentrations could indicate the euthyroid sick syndrome (ESS), which is frequently observed in patients with severe non-thyroid illness, such as DKA (23). For instance, in a recent retrospective research involving 163 T1DM cases, 60 (37.3%) presented ESS (24). Thus, we planned to measure reverse triiodothyronine (rT3), antithyroid peroxidase-antibody (TPO-Ab), anti-thyroglobulin antibodies (TG-Ab), thyroglobulin (TG), TSH-receptor antibody (TR-Ab), cortisol and corticotropin (ACTH) at 8.00, 16.00, and 24.00. However, the patient refused the mentioned blood tests. Thus, whether the patient was accompanied with central hypothyroidism or ESS remained unverified.

SCLC is an aggressive type of lung cancer with an estimated 250,000 new cases and over 200,000 deaths each year globally (25). China suffers a heavy burden of SCLC and an increasing trend for ICIs therapy. Tislelizumab has gained advantage due to lower financial burden but equivalent efficacy compared to other ICIs. Moreover, with specific design to minimize Fcγ receptor (FcγR) binding on the macrophages to impair antibody-dependent cellular cytotoxicity (ADCC) and antibody-dependent cellular phagocytosis (ADCP), tislelizumab showed better affinity to PD-1 and extremely lower off-rate compared to pembrolizumab and nivolumab (26, 27). Hence, as a relatively affordable ICI, tislelizumab has been widely accepted in SCLC first-line treatment, especially those with low or moderate income. With remarkable clinical benefit and acceptable adverse event in multiple solid tumors, tislelizumab has been approved by China, Food and Drug Administration (FDA) and European Medicines Agency (EMA).

Since the data of BGB-A317-312 has not yet been issued, we focused on published data of 934 cases from 4 clinical trials of single-agent tislelizumab, BGB-A317-001, BGB-A317-002, BGB-A317-203, and BGB-A317-204 (28–30). 5 cases (0.5%) were diagnosed as ICI-T1DM or hyperglycemia, with 1 (0.1%) grade 1 case, 1 grade 2 (0.1%) case, and 3 case (0.3%) were severe (grade ≥3), among which 2 severe cases were accompanied with DKA. The median onset time was 1.4 months (range: 1-10.5 months). 2 grade 1-2 cases returned to normal blood-glucose level, while 3 grade ≥3 cases need sustained insulin injection. 1 case (0.1%) temporarily withheld tislelizumab therapy and 1case (0.1%) permanently withdrew. 70 cases (7.5%) were diagnosed as hypothyroidism, with 19 (2.0%) grade 1 cases, 51 grade 2(5.5%), no grade ≥3 cases were observed. The median onset time was 3.5months (range: 0.7-24.1months). 2 cases (0.2%) temporarily withheld tislelizumab therapy and no case permanently withdrew. According to European society for medical oncology (ESMO) guideline of management of toxicities from immunotherapy, asymptomatic cases or with mild symptoms of hyperglycemia (new onset immune-related DM or worsening T2DM) can continue ICIs therapy with close follow-up and take insulin replacement as default therapy. In worsening T2DM, it is necessary to take dietary measures, optimization of oral hypoglycemic agents, and liaise with endocrinologist. In hyperglycemia case with moderate symptoms and no ketoacidosis, ICIs therapy should be considered to withhold until stabilized insulin replacement as default therapy, and hydration is clinically appropriate. Case with severe or life-threatening symptoms, such as ketoacidosis (grade 4), immediate medical attention and institutional management guidelines for DKA is required. ICIs therapy should be withheld until stabilized insulin replacement as default therapy. Similarly, according to the management of immune checkpoint inhibitor-related toxicities of National Comprehensive Cancer Network (NCCN) (version 1. 2024), urgent endocrine consultation, inpatient care, manage DKA as per institutional guidelines, and hold immunotherapy until DKA resolves are required in ICI-T1DM ketoacidosis case with low C-peptide level.

The present case suffered grade 4 irAE, and reached a stable blood glucose level with subcutaneous injection of insulin. However, the patient showed low compliance to glucose management, and even anti-cancer treatment. Even though loads of persuasion on glucose management including self-monitoring, regular insulin injection and consulting with endocrinologist has been made during hospitalization, he re-visited our clinic with hyperglycemia (random venous glucose: 55.08mmol/L) in a week. We believed that such hyperglycemia was entirely avoidable if continuous monitoring of glucose and insulin injection were proceeded as advised. Actually, he hardly checked his blood glucose and made dose adjustment of insulin after discharge. Thus, we suggested a high risk of hyperglycemia and lethal DKA recurrence in this case. Besides, CT scanning showed that a distinct regression of tumor after 3 cycles of tislelizumab plus cisplatin-etoposide treatment. Moreover, the tumor was still stable after anti-cancer treatment was withheld for 2 months. There was no denying that tislelizumab was effective and ICIs could be rechallenged after stabilized insulin replacement. However, due to mentioned poor adherence to glucose management, he would probably die from DKA instead of cancer itself. Hence, tislelizumab is not recommended in follow-up therapy in this case.

In conclusion, the present case described a case of tislelizumab-induced type 1 DKA in SCLC for the first time. Despite the infrequency of ICI-T1DM, it can be life-threatening when it is presented in DKA at the first referral. After 3 cycles of tislelizumab therapy, the present case already showed IFG, which was commonly observed in stage 2 T1DM. But we did not give enough consideration to elevated blood glucose, and finally lead to the occurrence of ketoacidosis. In this case, considering that the patient never took routine blood tests, quit of anti-cancer therapy not only reduce the lifetime of SCLC, but also increased the difficulty for glucose monitoring. Hence, patient education, regular glucose monitoring, intervention of high blood glucose, and withhold of ICIs treatment is crucial to avoid the onset of severe complications like ketoacidosis in stage 3 T1DM. We hope to draw a lesson from the present case, and put emphasis on the change of blood glucose, HbA1c and C-peptide during whole ICIs treatment course. Moreover, the establishment of individual health records for outpatient follow-up, lectures on mental and physical health worth further improvement.

## 4 Patient perspectives

Financial situation and education experience often influences the adherence to therapy. We believe that well-educated and high-income patients shows better treatment compliance compared to those with lower education and income level. Moreover, negative coping for terminal disease also contribute to the abandoning of anti-cancer therapy. Thus, individualized treatment plan based on patient’s financial situation is important to guarantee the compliance to finish anti-cancer therapy. Despite the patient refused further anti-cancer therapy, there was still a necessity for glucose monitoring to avoid the recurrence of lethal DKA. Early intervention of hyperglycemia, psychological support, health education, self-care awareness and self-management of patients, and routine consultation with an endocrinologist are also necessary to avoid lethal irAEs.

## 5 Limitations

Our study still had some limitations. Firstly, due to limited conditions, only IAA is routinely detected in our clinical laboratory. Thus, other autoimmune antibodies like islet cell antibody (ICA), glutamic acid decarboxylase (GADA), tyrosine phosphatase-like protein IA-2 (IA-2A), and zinc transporter 8 (ZnT8A) were not mentioned in the present study. More autoimmune antibodies might provide forceful evidence to the diagnosis of T1DM. Secondly, increased beta-hydroxybutyrate level (> 3mmol/L) strongly supported the diagnosis and severity assessment of DKA. However, blood beta-hydroxybutyrate level has not been routinely detected in our clinical laboratory. Thirdly, we have noticed the influence of hyperglycemia to C-peptide secretion and tried to monitoring C-peptide dynamically. However, the patient showed poor adherence to treatment and refused frequent blood tests. Thus, the only available C-peptide data was 5 days after hyperglycemic emergency. Consulting with an endocrinologist, health education of patients should be strengthened to avoid the mentioned deficiencies in our further research.

## Data Availability

The original contributions presented in the study are included in the article/**Supplementary Material**. Further inquiries can be directed to the corresponding authors.
